# A Probationary Essay on the Special Pathology of the Accessory Organs of Hearing; Submitted by the Authority of the President and His Council to the Examination of the Royal College of Surgeons of Edinburgh, When Candidate for Admission into Their Body, in Conformity to Their Regulations Respecting the Admission of Ordinary Fellows

**Published:** 1841-01

**Authors:** 


Art. V.—*A Probationary Essay on the Special Pathology of the Accessory Organs of Hearing; submitted by the Authority of the President and his Council to the examination of the Royal College of Surgeons of Edinburgh, when Candidate for admission into their body, in conformity to their Regulations respecting the Admission of Ordinary Fellows.* By James Mercer, m.d. &c.—*Edinburgh*, 1840. 8vo, pp. 133.

Had we ourselves never written any article on the subject of this pamphlet, and had we been ignorant of its literature, we might have awarded the author considerable praise; but, unfortunately for him, we have both written and redd on the subject, and we regret to be obliged to say that, in perusing his essay, we have met with very much that we had redd word for word before, and some things even which we ourselves have penned;—and all not only unacknowledged, but given as original. As one example of this plagiarism, we would refer to the sections on the anatomical structure of the membrana tympani, of the tympanic cavity, and of the Eustachian tube, which are almost wholly a reprint of the descriptions given by Mr. Wharton Jones in the article "Organ of Hearing" in the Cyclopædia of Anatomy and Physiology. The depredations committed on our own domain are not quite so extensive, but his drafts on Kramer are far from inconsiderable.

